# Phage susceptibility to a minimal, modular synthetic CRISPR-Cas system in *Pseudomonas aeruginosa* is nutrient dependent

**DOI:** 10.1098/rstb.2024.0473

**Published:** 2025-09-04

**Authors:** Josie F. K. Elliott, Keira Cozens, Yueyi Cai, Gretel Waugh, Bridget N. Watson, Edze Westra, Tiffany B. Taylor

**Affiliations:** ^1^ Milner Centre for Evolution, Department of Life Sciences, University of Bath, Bath BA2 7AY, UK; ^2^ ESI, University of Exeter – Cornwall Campus, Penryn TR10 9FE, Cornwall, UK

**Keywords:** CRISPR-Cas, bacteria–phage interactions, microbial ecology and evolution

## Abstract

CRISPR-Cas systems can provide adaptive, heritable immunity to their prokaryotic hosts against invading genetic material such as phages. It is clear that the importance of acquiring CRISPR-Cas immunity to anti-phage defence varies across environments, but it is less clear if and how this varies across different phages. To explore this, we created a synthetic, modular version of the type I-F CRISPR-Cas system of *Pseudomonas aeruginosa*. We used this synthetic system to test CRISPR-Cas interference against a panel of 13 diverse phages using engineered phage-targeting spacers. We observed complete protection against eight of these phages, both lytic and lysogenic and with a range of infectivity profiles. However, for two phages, CRISPR-Cas interference was only partially protective in high-nutrient conditions, yet completely protective in low-nutrient conditions. This work demonstrates that nutrient conditions modulate the strength of CRISPR-Cas immunity and highlights the importance of environmental conditions when screening defence systems for their efficacy against various phages.

This article is part of the discussion meeting issue ‘The ecology and evolution of bacterial immune systems’.

## 1. Introduction

Our knowledge of the range of systems that prokaryotes use to defend themselves from the viruses that infect them—bacteriophages (phages)—has rapidly expanded in the last decade [1]. In conjunction with this, more high-throughput studies are revealing the intricacies around which defence systems provide resistance to a host against which phage species [2]. Phages are highly diverse, such as in recognizing hosts through different receptors, carrying their own anti-defence systems, or replicating via different life cycles. Virulent phages undergo a lytic lifecycle and temperate phages are capable of either lytic or lysogenic lifecycles. In the lytic life cycle, phages use host machinery to lyse and kill the cell, and in the lysogenic life cycle phages incorporate into the host genome (where they are called prophages) to be replicated with the host genome, before excising to replicate and lyse the cell at some later time [3,4]. Owing to this diversity, no single defence system can offer protection to a host cell against all phages, and in turn it is likely that no phage can evade every defence system. As interest in using phages to control bacterial growth in medical or agricultural settings grows [5–7], an improved understanding of the traits enabling phages to evade bacterial defence systems, and whether any environmental factors can promote that evasion, will allow us to develop and apply more effective phage-based treatments.

One of the most well studied bacterial defence systems is the CRISPR-Cas (clustered regularly interspaced short palindromic repeats—CRISPR-associated) system (reviewed [8]). Upon entry to the cell, Cas (CRISPR-associated) proteins select, process and integrate regions of the invading DNA (called protospacers) into the CRISPR array (reviewed in [9]). Upon incorporation, the unique DNA sequences are called spacers, and the immunity they provide can be inherited. CRISPR arrays can contain many spacers, targeting the same or different mobile genetic elements (MGEs), and hosts can contain multiple CRISPR arrays [10]. This process of gaining new spacers is called adaption or spacer acquisition. Each spacer is transcribed and processed to produce a crRNA (CRISPR RNA), which then forms ribonucleoprotein complexes with Cas proteins. Through complementary base pairing, the spacer sequences guide Cas nucleases to degrade targeted genetic material, providing immunity to the host cell [8]. This process of targeting, binding and nuclease-mediated destruction of invading genetic material is termed interference.

In type I CRISPR-Cas systems, acquisition of new spacer sequences into the CRISPR array can occur via either naive or primed acquisition [9]. Naive acquisition is as described above, whereas primed acquisition occurs when there are pre-existing (priming) spacers that may have complete or partial complementarity to the re-infecting foreign genetic material. These ‘priming’ spacers are able to enhance the acquisition of new spacers [11–13].

Although in theory a CRISPR-Cas system could acquire spacers and target any incoming genetic element, the capabilities of CRISPR-Cas systems as phage defences are more curtailed in nature. Computational predictions have suggested that hosts with CRISPR-Cas systems may rarely outcompete those lacking the system during virulent phage epidemics, and during prophage epidemics hosts with CRISPR-Cas systems are only successful when the prophage incurs considerable cost to the host [14]. Previous studies have navigated the challenge of natural acquisition of de novo CRISPR-Cas immunity by engineering species to insert spacers against phages to study functional aspects of the CRISPR-Cas system [15–17]. Interestingly, in these engineered strains, it is observed that CRISPR-Cas systems do not always offer protection and benefit to the host. In *Escherichia coli*, when the type I-E CRISPR-Cas system was directed against a variety of lytic phages, only spacers targeting pre-early genes (which transcribe and shut down host cellular functions before the entire phage genome enters [18]) were able to elicit protection against the T5 phage. Against the T4 and R1-37 phages CRISPR-Cas was ineffectual at providing protection, owing to an unknown CRISPR-Cas interference phage evasion mechanism [19].

In some cases the inability of CRISPR-Cas systems to provide protection against phage lysis to their hosts can be explained by factors such as anti-CRISPR proteins (Acrs) that block CRISPR-Cas activity [20], or the proteinaceous nucleus-like structures some jumbo phages use to shield their genetic material from CRISPR-Cas systems [21] or alternatively the genetic material of phages can be disguised using epigenetic DNA modifications [22]. In other cases, CRISPR-Cas immunity has been suggested to be too slow to enable recovery from infection from virulent phages, resulting in an abortive infection phenotype [23]. However, it is still unclear if the ecological advantage of a CRISPR-Cas system against any phage species may be predicted by traits of the phage.

Furthermore, it is yet unknown how factors that result in imperfect protection by CRISPR-Cas systems against phage replication may depend on ecology. The effect of environmental parameters on CRISPR-Cas evolution has been explored in previous work on *P. aeruginosa* UCBPP PA14 with its lytic phage DMS3vir (which is a mutant of the temperate phage DMS3 locked into the lytic lifestyle [24,25]). The preference of populations to evolve CRISPR-Cas immunity via spacer acquisition, rather than via selection that enriches surface receptor mutants that prevent phage-binding (the type IV pilus in the case of DMS3vir), is highly sensitive to both biotic and abiotic factors. For example, the evolution of DMS3vir phage resistance via CRISPR-Cas spacer acquisition was favoured in low-nutrient conditions [26], which may be due to increased mutational supply in high-growth conditions as well as selection for CRISPR immunity over surface mutants under low phage densities [27]. Similarly, increased CRISPR-Cas adaptation was observed when PA14 populations were grown at lower-than-optimal temperatures [28]. Slowed growth in the presence of bacteriostatic antibiotics has also been shown to increase spacer acquisition and favour CRISPR-Cas immunity [29]. Similarly, translation-inhibitory antibiotics increase CRISPR immunity levels against phages that carry Acrs [30]. This suggests that CRISPR-Cas systems may present a more formidable barrier to phage infection in limited resource environments. However, the *P. aeruginosa* model has only been monitored against DMS3vir or closely related phage [31], thus it is still unknown if the ecological sensitivity applies to a broader panel of phages. Additionally, it is unclear the influence that ecological conditions may have on CRISPR-Cas interference only, as opposed to the evolution of immunity via spacer acquisition.

To address this, we created a synthetic, minimal version of the type I-F CRISPR-Cas system from *P. aeruginosa,* made modular with restriction enzyme recognition sites for easy exchange of genetic elements such as spacers. The synthetic CRISPR-Cas system allowed us to engineer strains with complementary spacers to a panel of phages with various characteristics and experimentally test whether CRISPR-Cas interference is equally effective against a diverse panel of phages, and whether the efficacy of CRISPR-Cas interference increases under low-nutrient conditions.

## 2. Material and methods

### (a) Bacterial strains and phages

All strains cloned in this study were created from the genetically engineered ΔCRISPR-Cas version of the UCBPP-PA14 strain, described herein as ‘CRISPR-Cas knock-out’ [32]. The synthetic CRISPR-Cas system was validated primarily using the phage DMS3vir, which was engineered from the phage DMS3 to be obligately lytic [24]. During the efficiency of plating experiment, the related phages were used: DMS3mvir-AcrIF1 (which contains a type I-F-targeting anti-CRISPR gene) and DMS3mvir-AcrIE3 [20]. The CRISPR-Cas system was screened against the *P. aeruginosa* temperate/lysogenic phages detailed in electronic supplementary material, table S1.

All overnight cultures were incubated at 37°C, shaking at 200 r.p.m. in LB (Miller’s lysogeny broth) or M9 medium (47 mM Na_2_HPO_4_; 22 mM KH_2_PO_4_; 8.6 mM NaCl; 20 mM NH_4_Cl; 1 mM MgSO_4_; 0.1 mM CaCl_2_) supplemented with 0.2% glucose.

### (b) Cloning synthetic CRISPR-Cas system into *Pseudomonas aeruginosa* using mini-Tn7 insertion

**Figure 1.**
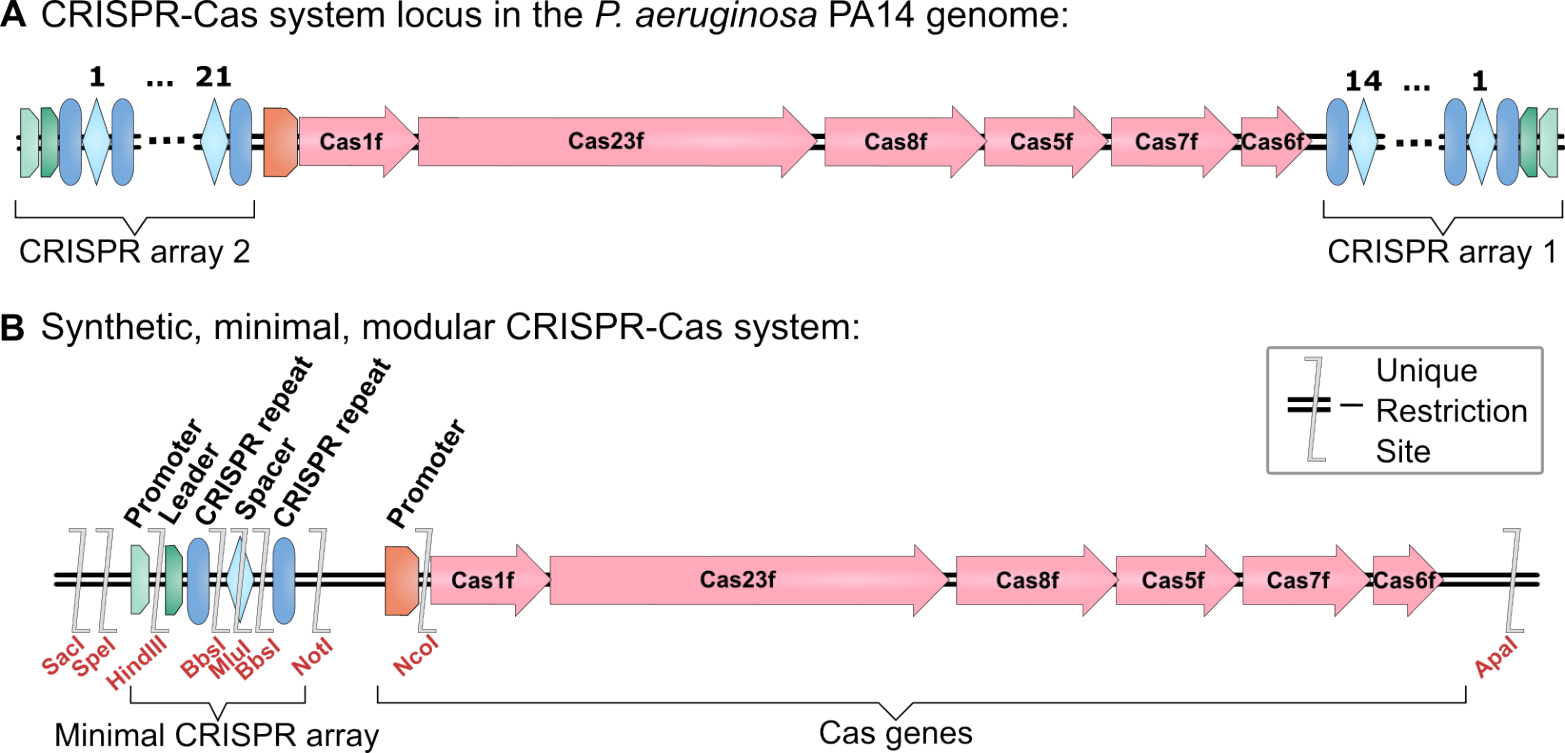
Validation of a synthetic version of the type I-F CRISPR-Cas system of *Pseudomonas aeruginosa* PA14. (A) Schematic of the CRISPR-Cas system locus in the genome of *P. aeruginosa* PA14 (adapted from [24]). This contains two CRISPR arrays, and six *cas* genes: Cas1f: endonuclease Cas1; Cas23f: helicase also known as Cas3; Cas8f: also known as Csy1; Cas5f: also known as Csy2; Cas7f: also known as Csy3; Cas6f: endoribonuclease also known as Cas6/Csy4 (names updated from the [24] in line with naming conventions in [33]). In the genome, this locus is antisense but has been displayed in the same sense orientation as the synthetic CRISPR-Cas system for ease of comparison. The relative lengths of each gene in the schematic have been drawn proportional to sequence length. (B) Schematic of the synthetic, minimal, modular CRISPR-Cas system which has the CRISPR arrays reduced to one repeat–spacer–repeat from the CRISPR 2 array. The relative positions of the added restriction sites are shown.

The synthetic CRISPR-Cas DNA, obtained from GeneArt Thermofisher Scientific (https://benchling.com/s/seq-gNNEFhMwdBo2URdFk7Y8), was based on the *P. aeruginosa* PA14 type I-F CRISPR-Cas system, minimized for functionality, with restriction sites added for modularity (figure 1). The CRISPR array contains a single repeat–spacer–repeat sequence from the CRISPR 2 array (shown to acquire new spacers more frequently [28,34]). The leader sequence of the CRISPR 2 array was annotated as the 134 base pairs upstream of the first CRISPR repeat (sequence supplied by Vorontsova *et al*. [17]). The promoter was assumed to be contained within 135 bases upstream of the leader sequence and downstream of the adjacent gene *PA14-3370*. The non-targeting spacer, with two *Bbs*I recognition sites and a MluI site for modularity, was designed that had no targets in the *E. coli* or *P. aeruginosa* chromosomes. A strong rrnB T1 terminator was added downstream to prevent read-through from the CRISPR array [35], flanked by *Nsi*I restriction sites. The *cas* operon promoter, assumed to be between the CRISPR 2 array and *cas1* genes, was made interchangeable with *Not*I and *Nco*I sites. Other restriction enzyme recognition sites (*Bbs*I, *Mlu*I, *Nsi*I, *Not*I, *Nco*I and *Asc*I) were then edited out using *P. aeruginosa* codon usage tables [36,37].

The synthetic CRISPR-Cas DNA was transferred into the pUC18T-mini-Tn7T-Gm plasmid (pUC18T-mini-Tn7T-Gm was a gift from Herbert Schweizer—Addgene plasmid no. 63121; http://n2t.net/addgene:63121; RRID:Addgene_63121) [38] via restriction–ligation cloning using *Apa*I and *Spe*I (New England Biosciences). To incorporate new spacers against either plasmid or phage, the spacer sequences were ordered as two overlapping primers which, when annealed, create sticky ends compatible with the *Bbs*I restriction sites. Oligonucleotides were annealed by heating in annealing buffer (100 mM potassium acetate, 30 mM HEPES, pH 7.5) at 95°C for 5 min before slowly cooling (30 s 0.5°C stepwise cooling to room temperature). The spacer DNA was phosphorylated with T4 polynucleotide kinase before ligation with the pUC18T-mini-Tn7T-Gm-CRISPR-Cas plasmid (digested with *Bbs*I and dephosphorylated with shrimp alkaline phosphatase (New England Biosciences)). Successful insertion was determined by polymerase chain reaction (PCR) with a forward primer against the pUC18T-mini-Tn7T-GM plasmid (Tn7-F; electronic supplementary material, table S2) and a reverse primer after the CRISPR array (CRISPR-R; electronic supplementary material, table S2), then confirmed via Sanger sequencing (Source Bioscience).

The synthetic CRISPR-Cas DNA was inserted as a single copy into the chromosome of *P. aeruginosa* CRISPR-Cas knock-out using the four-parent conjugal puddle mating conjugation version of the protocol from Choi & Schweizer [38]. The Tn7 transposon integrates at the single *att*-Tn7 downstream of the *glmS* gene in the *P. aeruginosa* genome. PCR (glmS-F and CRISPR-R primers; electronic supplementary material, table S2) was used to verify insertion.

To create phage-targeting spacer strains, oligonucleotides of two spacers (separated by a repeat sequence), targeting different regions in the phage genome were cloned as described above into the synthetic CRISPR-Cas system (electronic supplementary material, table S3). The type I-F CRISPR-Cas system requires a protospacer adjacent motif (PAM) sequence of ‘GG’ in the targeted genome. Previous research has identified the linear genome ends as protospacer sampling hotspots [39]; therefore, spacer 1 targeted gene regions near phage genome ends (and these regions were verified by PCR and Sanger sequencing of the phage). Spacer 2 targeted identified genes in the central region of the genome.

### (c) One-step phage growth curves

*Pseudomonas aeruginosa* wild-type cultures were grown overnight in LB then adjusted to OD_600_ of 2.6 in 1 ml LB and inoculated into 25 ml LB in a 250 ml flask to give a starting OD_600_ of 0.1. Cells were grown with shaking at 37°C to early exponential phase (OD_600_ 0.2−0.3), at which point 100 mμl of phage at approximately 10^9^ (plaque-forming units (pfu) ml^−1^) was added. This gave a starting phage titre of approximately 4 × 10^6^ pfu ml^−1^ and starting multiplicity of infection (MOI) of approximately 0.01. Samples were taken at various time points up to 120 min post infection and added to phosphate-buffered saline (PBS) and chloroform (sample : chloroform 10 : 1 v/v) to lyse cells, allowing measurement of the total number of mature phages. Samples were serially diluted, and 2 μl was spotted in triplicate on a lawn of wild-type *P. aeruginosa* PA14. The phage PA14P2 had to be grown and plated on *P. aeruginosa* PA14 csy3::LacZ as the wild-type CRISPR array carries a spacer against this phage. Owing to sampling only chloroform average, burst size per cell was calculated from the number of phages released at the maximum of the growth curve, (max. pfu ml^−1^ – min. pfu ml^−1^)/the number of cells infected (*t* = 0 pfu ml^−1^ – min. pfu ml^−1^), giving the total number of new phages in the population per infected cell. Phage adsorption over time was calculated relative to the eclipse period as the number of phages lost from the medium up to the minimum of the growth curve (*t* = 0 pfu ml^−1^ – min. pfu ml^−1^)/(*t* = 0 pfu ml^−1^). The eclipse period (period from phage infection to maturation of new phage particles) was defined at the minimum of the growth curve before the phage burst starts [40].

### (d) Phage virulence assays

The method to determine phage virulence was adapted from the metrics in Storms *et al*. [41]. Overnight cultures of wild-type *P. aeruginosa* (and csy3::lacZ for PA14P2 phage) were grown in 10 ml LB then normalized to OD_600_ of 1. Each culture was resuspended in PBS and added to sterile LB in 96-well plates at 1 : 100 dilution, and growth curves were performed in biological and technical triplicate. Phages were diluted from the highest harvestable titre (to a maximum of 10^9^ pfu ml^−1^) by a factor of 10 across five dilutions and added 1: 100 into the wells in addition to a PBS no-phage control. Phage and bacterial titres were also plated and measured at the start of the experiment to allow calculation of accurate MOI. OD_600_ measurements were taken every 10 min for 23 h while the plate was incubated at 37°C with shaking at 180 r.p.m.

To prevent the selection for phage-resistant cells impacting the calculated virulence index, the growth curve measurements were cut off at the time at which the average OD_600_ readings for the samples with the highest concentration of phage surpassed the average OD_600_ readings for the second highest concentration of phage (or the third highest if the first two concentrations could not be resolved). Local virulence was calculated as: 1 − (area under the curve at each given MOI/area under the curve at no-phage dilution). Virulence index was then calculated from local virulence as the area under the local virulence curve divided by the theoretical maximum area under the virulence curve (area if local virulence equalled 1 for all MOI).

### (e) Bacterial growth curves

Overnight cultures of each strain were grown in 10 ml of LB then normalized to OD_600_ of 1. Each culture was resuspended in PBS and added a 1: 100 dilution to LB or M9 in sterile 96-well plates. Phage cultures were diluted in PBS and added at 1 : 100 to the wells to give each concentration. OD_595_ measurements were then taken every 10 min for 23 h while the plate was incubated at 37°C, shaking at 180 r.p.m. Phages were added at two concentrations: high (10^8^ pfu ml^−1^ then diluted 1 : 100 into 96-well plate) and low (10^6^ pfu ml^−1^ then diluted 1 : 100 into 96-well plate), as well as PBS being added for the no-phage control.

### (f) Statistics

All statistical analysis were performed in the R statistical package 4.4.0 (last accessed 24 April 2024) [42]. Data that ranged over three powers of ten or more were log transformed to normalize residuals. Statistical significance for the efficiency of plating and conjugation efficiency was determined using one-way ANOVA analysis with *post hoc* analysis via Tukey test. Pairwise comparisons depicted on graphs were *t*-tests, any *p*-value adjustments being detailed in figure legends. Generalized linear mixed effects models were fitted to the data to determine whether any of the measured characteristics significantly predicted sensitivity to CRISPR-Cas interference. To test for correlation between phylogenetic relatedness and sensitivity to CRISPR-Cas interference, *δ* [43] was calculated independently for phylogenetic trees based on the terminase large subunit (TerL) and the major capsid protein.

## 3. Results

### (a) A minimal, modular, synthetic CRISPR-Cas system allows a functional, genetically tractable defence system in the host genome

**Figure 2.**
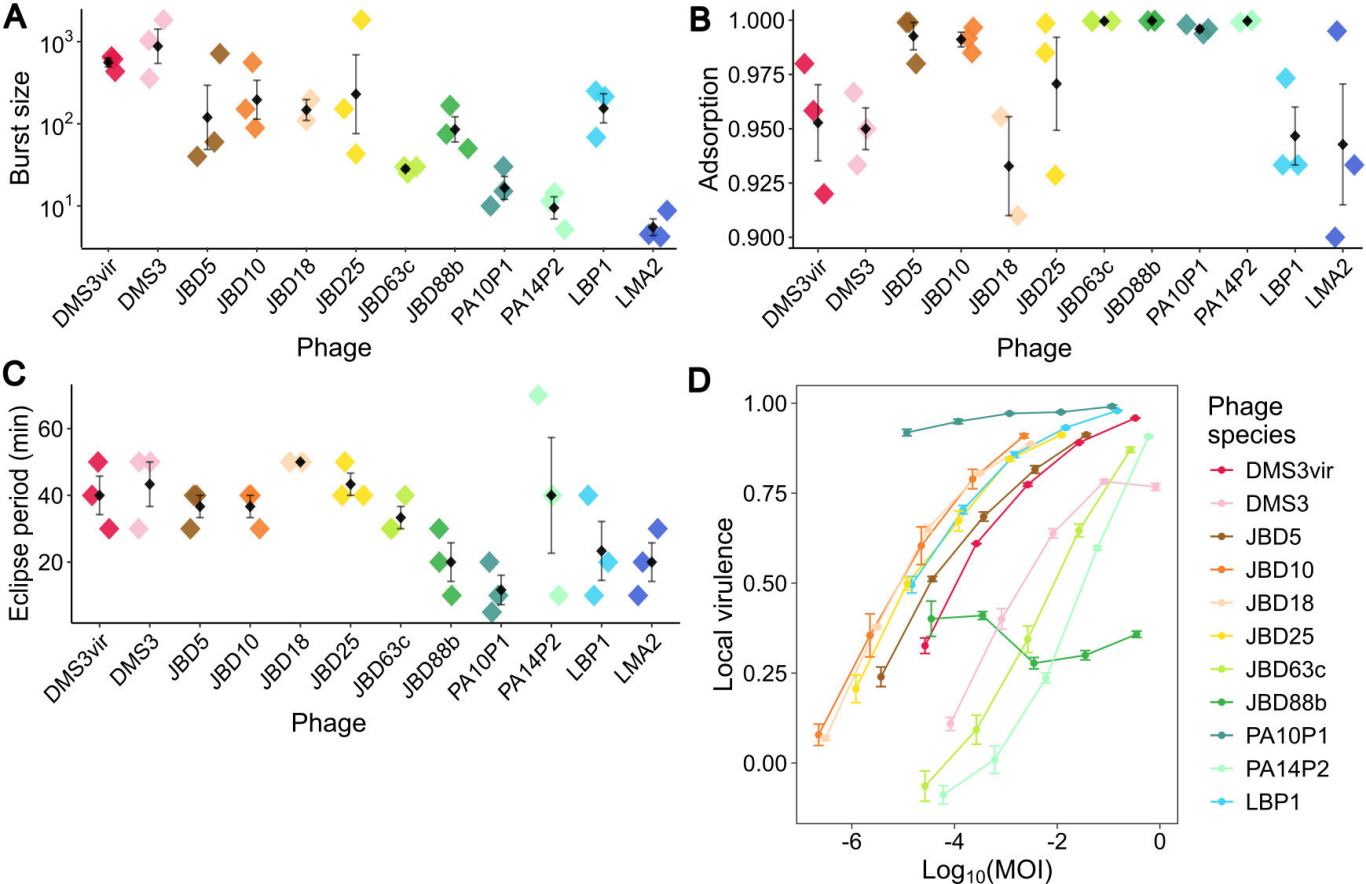
Comparison of the infection characteristics of a panel of 13 phages. Each phage is presented in the same colour across each panel. Assays were performed on wild-type *P. aeruginosa* strains (apart from PA14P2 phage, which used a *csy3*::lacZ knock-out strain). (A–C) Black diamonds represent mean values (*n* = 3) and error bars show the standard error around the mean. Diamonds coloured by phage show individual replicates. (A) Burst size represents the number of new phage particles released per cell during the burst phase of the one-step growth curve when the first mature phages are assembled post infection. The growth cycle for PA14P2 extended beyond the 2 h measurement window of this experiment; thus the average burst size calculated for this phage is likely an underestimation. (B) Adsorption describes the fraction of total phage population taken up by the cells in the medium during the first part of the growth cycle. (C) Eclipse period describes the time taken from initial infection to when the first mature phages start to be assembled. (D) Plot of local virulence across log multiplicity of infection (MOI). Circles represent mean values (*n* = 3), and error bars show standard error around the mean. Local virulence represents the area under a growth curve at each MOI of phage infection, normalized against the growth curve when no phages are present.

We sought to create a version of a CRISPR-Cas system within which all the components can be easily edited. This would be a potentially useful tool for studying how the mechanisms of CRISPR-Cas systems impact co-evolution with phages. The type I-F CRISPR-Cas system of *P. aeruginosa* UCBPP-PA14 contains two CRISPR arrays (CRISPR 1 and CRISPR 2) which flank six *cas* genes [24,33] (figure 1A). This spans around 14 kb in the genome. A synthetic version of this CRISPR-Cas system was designed to be minimal, modular and genetically tractable (figure 1B). The two CRISPR arrays were reduced to a single array with a single spacer flanked by repeats. The system is also made modular by the addition of restriction endonuclease (RE) recognition sites flanking key regions (figure 2B). The type-II RE recognition sites within the spacer face outwards, allowing scarless replacement of the spacer with any number or design of spacers using either short oligonucleotides with simple restriction ligation cloning, or multiple fragments using golden gate cloning as has been used to produce multi-spacer guide RNAs [44]. Thus, the synthetic CRISPR-Cas system can be quickly and easily edited to target any required sequence that has suitable protospacer adjacent motif recognition sites.

This synthetic CRISPR-Cas system was stably integrated as a single copy into the host chromosome using a Tn7 transposon-based method, eliminating the need for plasmid maintenance and consistent antibiotic selection, thus making it ideal for long-term evolution experiments. Furthermore, this avoids dosage variability that may be associated with plasmid copy number. The insertion of the synthetic CRISPR-Cas system into the *P. aeruginosa* CRISPR-Cas knock-out strain did not result in a fitness cost to the host (electronic supplementary material, figure S1A) and includes a selectable gentamicin resistance marker for easy identification of edited strains. The synthetic CRISPR-Cas system was shown to be capable of specifically targeting plasmids (electronic supplementary material, figure S1B), was able to target the DMS3vir phage at similar efficiencies to the native CRISPR-Cas system (electronic supplementary material, figure S1C) and was shown to be capable of acquiring spacers via both primed and naive acquisition (electronic supplementary material, figure S1D). Taken together these results show that the synthetic CRISPR-Cas system retains all activities of the native CRISPR-Cas system when complemented into *P. aeruginosa*.

### (b) The panel of phages used to test CRISPR-Cas interference efficacy possessed a broad range of infectivity metrics

We initially hypothesized that the efficacy of CRISPR-Cas interference may vary with traits associated with phage biology such a virulence, e.g. CRISPR-Cas immunity may be more effective against less virulent phage. We therefore selected a panel of 13 diverse double-stranded DNA (dsDNA) phages (dsDNA being the most sampled phage type), including those with lytic and lysogenic lifecycles and that use different surface receptors, to test whether CRISPR-Cas interference and ability to provide protection against phages is dependent on any predictable characteristics of a phage (electronic supplementary material, figure S2). Included were virulent phages PA10P1, PA14P2, LMA2, PhiKZ; temperate phages DMS3, JBD5, JBD10, JBD88b, JBD63c, LBP1, JBD18, JBD25; and the non-lysogenic mutant DMS3vir. The phages DMS3vir, DMS3, JBD5, PA14P2, JBD63c, LBP1, JBD18, and JBD25 all use the type IV pilus (T4P) as a receptor, JBD88b and PA10P1 use the lipopolysaccharide (LPS), JBD10 has been found to target both, and LMA2 is hypothesized to target the TonB receptor. PhiKZ is a jumbo phage previously shown to resist CRISPR-Cas immunity via a proteinaceous shell around its DNA [45] and thus served as a negative control. To quantify the characteristics of these phages, we performed one-step growth curve and phage virulence experiments (figure 2).

The one-step growth curve assay standardizes the start of the phage infection cycle for all phages in a sample, allowing measurements of how many phages are released per cell (burst size, figure 2A), what fraction of the phage population successfully adsorb to and inject their DNA into cells (adsorption, figure 2B), and how long the phage replication cycle takes until mature phages are assembled (eclipse period, figure 2C). The plaques from jumbo phage PhiKZ on solid media were not able to be resolved owing to their hazy morphology; thus PhiKZ was excluded from this experiment. The remaining phages showed a range of phenotypes that vary between infection metrics, e.g. the phage with the highest burst size—DMS3—does not have the highest adsorption or shortest eclipse period. Each individual growth curve is shown in electronic supplementary material, figures S5–S17A. A secondary test of adsorption kinetics of only free phage (non-chloroformed samples) was also performed (electronic supplementary material, figure S3). Together, this panel of phages thus represented a range of infectivity metrics, allowing us to test CRISPR-Cas interference against phages with a range of lifestyles, receptors and virulence.

**Figure 3.**
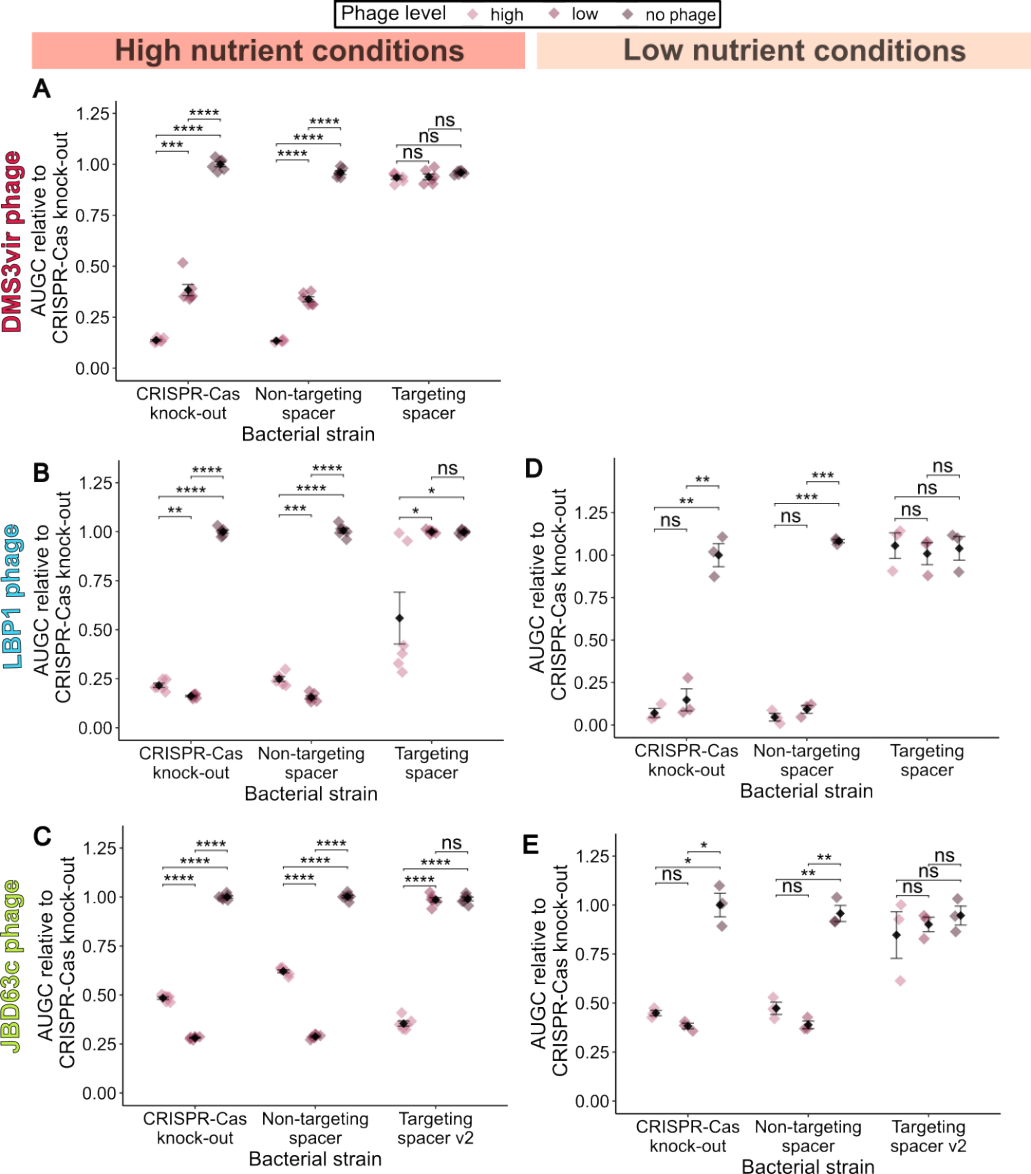
Growth dynamics in response to the various phages with and without a CRISPR-Cas system. Area under growth curve (AUGC) was normalized relative to the average AUGC for the CRISPR-Cas knock-out strain without phages added and plotted for each strain. The black diamond shows the mean (*n* = 6 for high-nutrient condition experiments and *n* = 3 for low-nutrient condition experiments) for each strain, with error bars representing the standard error around the mean. Diamonds for each individual datapoint are coloured by the phage level (displayed left to right: high phage MOI 0.01, low phage MOI 0.0001, no-phage MOI 0). Within each strain, the means at each phage level were compared with *t*-tests with false discovery rate (FDR) *p*-correction (*p < 0.05, **p < 0.01, ***p < 0.001, ****p < 0.0001, ns: non-significant). (A) AUGC plot against bacterial strain with high, low or no DMS3vir phage added in high-nutrient conditions. (B) AUGC plot against bacterial strain with high, low or no LBP1 phage added in high-nutrient conditions. (C) AUGC plot against bacterial strain with high, low or no LBP1 phage added in low-nutrient conditions. (D) AUGC plot against bacterial strain with high, low or no JBD63c phage added in high-nutrient conditions. (E) AUGC plot against bacterial strain with high, low or no JBD63c phage added in low-nutrient conditions.

The ability of each phage to infect and prevent growth of the host bacteria was assessed using a virulence assay in liquid culture (figure 3D). In this assay, phages are diluted to give a range of phage-to-cell ratio values and the effect on growth is measured via a growth curve. To produce the metric of ‘local virulence’ the area under the growth curve at each phage dose is normalized against the area under the growth curve in the absence of phages. Individual growth curves at each phage dose are shown in electronic supplementary material, figures S5–S17B. The area under the local virulence curve can also be calculated and normalized against the maximal possible area to give an individual value termed the 'virulence index' (electronic supplementary material, figure S2). The higher the local virulence the more virulent the phage, with a maximum value of 1 if there is no growth in the presence of the phage. PhiKZ was excluded as it could not be grown to a high enough titre to give at least three log_10_ dilutions that impacted growth. The LMA2 phage was also excluded as it elicited a bacterial precipitate when cultured in liquid, which interfered with the spectrophotometer OD_595_ readings. Of the 11 phages tested, there is a clear diversity in phage virulence from the most virulent PA10P1 to the least virulent PA14P2 and JBD88b.

### (c) CRISPR-Cas can provide complete protection against most phages but only nutrient-dependent protection against others

The ease with which the spacer in the synthetic CRISPR-Cas system can be interchanged with any phage-targeting sequence desired presented an opportunity to test CRISPR-Cas interference against each of the 13 phages. Synthetic CRISPR-Cas *P. aeruginosa* strains were created that had two spacers designed to target different regions of the genome in each phage (electronic supplementary material, table S3). This ensured that the ability of the phages to evolve escape mutations against both spacers over the experimental period and confound results would be near impossible. Each spacer 1 in the phage-targeting strain CRISPR-Cas array was also validated by testing the ability of the CRISPR-Cas system to reduce conjugation efficiency when a short region of the phage genome containing the protospacer was cloned into a plasmid (electronic supplementary material, figures S5–S12D). These phage-targeting synthetic CRISPR-Cas strains were then tested to see if they could restore normal growth in the presence of each phage. The synthetic CRISPR-Cas system was able to provide complete immunity to eight of the phages tested: restoring the growth in liquid high-nutrient medium to similar levels to when phages were absent (DMS3vir, DMS3, JBD5, JBD10, JBD88b, PA10P1, PA14P2 and LMA2 (electronic supplementary material, figures S5–S12E)). Data for the DMS3vir phage are given in figure 3A as a representative example of the results obtained for these eight phages. For each phage, this phenotype was also confirmed on solid media using an efficiency of plating assay (electronic supplementary material, figures S5–12C). This use of the synthetic CRISPR-Cas system demonstrates that interference by the CRISPR-Cas machinery can provide protection against both virulent and temperate phages, across phages with a range of virulence strength, that target different receptors and against phages with a range of infection dynamics such as adsorption time, burst size and eclipse period (electronic supplementary material, figures S2 and S3). Linear regression was used to test whether the measured phage life history traits significantly predicted susceptibility. Linear mixed-effect models were fitted to the data with susceptibility as a dependent variable; each measured phage life history trait as a predictor, and replicate as a random effect. The models indicated that none of the traits significantly predicted susceptibility to CRISPR-Cas interference (electronic supplementary material, table S4).

We were able to observe that the phenotypic diversity of the phage panel was reflected in the genetic diversity of the phage used and this was also not correlated with susceptibility to CRISPR-Cas interference. Phylogenetic trees of all complete phage genomes labelled as targeting *P. aeruginosa* in the NCBI database (electronic supplementary material, figure S4) were reconstructed based on the amino acid sequence of both the terminase large subunit protein (electronic supplementary material, figure S4A) and the major capsid protein (electronic supplementary material, figure S4B) to ensure all 13 phages in our panel were included. Phylogenetic *δ* quantifies the strength of phylogenetic signal for categorical traits, where low values (approx. 0) indicate no correlation between phylogeny and trait, and high values indicate correlation between trait similarity and phylogenetic relatedness. For the major capsid protein phylogeny, *δ* was calculated as 0.025 (*p*‐value = 0.178), and as 2.26 for the terminase large subunit (TerL; *p*‐value = 0.15), indicating that there is no significant phylogenetic relationship for susceptibility or resistance to CRISPR-Cas interference.

For two phages, LBP1 and JBD63c, CRISPR-Cas interference with two phage-targeting spacers only produced an ‘intermediate’ protective phenotype (figure 3B,C, respectively). For the strains of *P. aeruginosa* with a CRISPR-Cas system containing a phage-targeting spacer, in the presence of low levels of either LBP1 phage or JBD63c phage, the CRISPR-Cas system was able to restore bacterial growth levels to the equivalent growth in the absence of phage. However, at high levels of phage, growth levels were reduced. In solid medium experiments, the CRISPR-Cas system was able to produce a significant, but small in magnitude, decrease in efficiency of plating (electronic supplementary material, figures S13–S14C). For three phages JBD18, JBD25 and PhiKZ, the addition of a CRISPR-Cas system appeared to have no effect on phage infection (electronic supplementary material, figures S15–S17).

For the five phages where the initial phage-targeting strains were not able to restore normal growth in nutrient-rich medium in the presence of phages—LBP1, JBD63c, JBD18, JBD25 and PhiKZ—a second version of the phage-targeting strain was created to ensure lack of complete phage defence was not due to poor spacer choice. These second-version spacers targeted new regions on the phage genome. CRISPR-Cas systems were tested via the same assays for ability to provide immunity to the host. For all these spacers that did not successfully target phage, the spacers in the synthetic CRISPR-Cas system were still able to successfully target the protospacer DNA when it was instead incorporated into a plasmid (electronic supplementary material, figures S13–S17D). This showed that the observed phenotype was due to some aspect of the phage, rather than anything intrinsic to the selected spacer sequence.

Slowed growth, such as through reduced nutrient concentrations, has previously been shown to favour the evolution of CRISPR-Cas in *P. aeruginosa* [26]. In light of this, for all phages against which possession of a CRISPR-Cas system did not completely restore the growth of the host, we repeated these liquid growth experiments in low-nutrient medium. In the presence of both LBP1 and JBD63c, strains with a phage-targeting spacer were then able to provide complete immunity in the presence of both low and high phage levels (LBP1: figure 3D; JBD63c: figure 3E). We therefore demonstrate that nutrient conditions can modulate the efficacy of specifically the interference step of CRISPR-Cas immunity against some phage. The genomes of both phages that showed nutrient-dependent sensitivity to CRISPR-Cas interference also possessed predicted hits for anti-CRISPR genes, although this was not unique to these two phages (electronic supplementary material, figure S2). We did not test other phages (including Acr-containing phages) under low-nutrient conditions. For phages where bacterial growth in the presence of CRISPR-targeting spacers was already indistinguishable from the no-phage control in high-nutrient conditions, we would not expect further changes in low-nutrient conditions, as there would be no scope for additional CRISPR-mediated protection.

Interestingly, for the JBD63c phage, one phage-targeting spacer strain did not provide any protection; however, for the second version of the phage-targeting strain, which used a different spacer sequence, the CRISPR-Cas system was able to provide intermediate protection in high-nutrient conditions and complete protection in low-nutrient conditions (electronic supplementary material, figure S14E,F). This shows that spacer choice can also impact the success of CRISPR-Cas interference even when the targeted genome contains an appropriate PAM sequence. Lack of effective interference has previously been shown in phages with pre-early genes [19,46]. The phages JBD18 and JBD25 showed complete resistance to CRISPR-Cas interference, including when experiments were repeated with new phage-targeting spacers and in low-nutrient conditions (electronic supplementary material, figures S14E,F and S15E,F, respectively), despite these spacers successfully targeting plasmids (electronic supplementary material, figures S14, S15D). However, given the demonstration by the JBD63c phage that successful CRISPR-Cas interference can be spacer-dependent, and that only two spacer variations of the synthetic CRISPR-Cas system in *P. aeruginosa* were tested, it remains likely that another spacer could yield immunity to these phages. The phage PhiKZ also showed complete resistance to CRISPR-Cas interference irrespective of alternative spacers or changing nutrient conditions (electronic supplementary material, figure S17). This was expected as PhiKZ is a jumbo phage that builds a proteinaceous nucleus-like structure around its DNA [47]; such structures have been shown to make the phage resistant to type I-F CRISPR-Cas systems [21].

Together these results show that upon acquiring spacers against a phage, CRISPR-Cas systems can provide robust immunity against a wide variety of phages. However, interference is not perfect, being modulated by the choice of acquired spacer (placing another barrier to successful immunity if not only must a cell acquire new spacers, but those spacers can only be from certain regions of the phage genome). Furthermore, we show for the first time to our knowledge that selection for CRISPR immunity varies across different environments owing to variation in the strength of resistance (CRISPR interference).

## 4. Discussion

CRISPR-Cas systems are unique in their ability to provide adaptive, heritable immunity to their hosts by the acquisition of spacers derived from invading genetic material paired with the sequence-guided destruction of matching DNA through nuclease-mediated interference. Although clearly offering an advantage, CRISPR is not ubiquitously distributed across bacterial genomes [48]. Furthermore, the evolution of phage protection via mutation of surface receptors targeted by phages is frequently shown to be favoured in populations over the evolution of CRISPR-Cas immunity via spacer acquisition [26,49]. As the repertoire of known defence systems and subtypes of these defence systems expands, there is increasing interest in an ability to map and predict which defences are robust against which phage [1,2], and this will be important in the development of ‘evolution-proof’ phage-based therapeutics [5].

Understanding the molecular details of phage and CRISPR-Cas system co-evolution will be aided by genetic tools that allow the precise dissection of the molecular components of the CRISPR-Cas system. To address this, we adapted the type I-F CRISPR-Cas system of *P. aeruginosa* to create a synthetic, minimal, modular CRISPR-Cas system where each element can be replaced by restriction–ligation cloning in *E. coli* and the whole system can then be stably integrated into the host genome using a highly repeatable Tn7 transposon-based system [38]. For example, strains with any desired spacer sequence or number of spacers can be created with inexpensive oligonucleotides in 1−2 weeks [44], and there is the potential also to change promoter sequences or integrate other genetic sequences alongside the CRISPR-Cas system. This contrasts with previous studies where CRISPR-Cas systems have had to be exposed to plasmids [50], phages [51] or libraries of phage fragments [19] in plasmids, from which acquisition events are detected and selected for. Other studies that have probed the molecular mechanisms of the *P. aeruginosa* type I-F CRISPR-Cas system have hosted *cas* genes and CRISPR arrays on plasmids [17]. In this study, the mini-Tn7 transposon-based integration system allows the CRISPR-Cas system to be stably inserted in the genome at a site-specific intergenic region, thus removing the requirement for continual selection for plasmid maintenance, and the potential effect of plasmid dosage as an extraneous variable. The Tn7 system can be applied to any host with an *att-Tn7* site in the genome [38,52], making this system applicable for integration of the synthetic CRISPR-Cas system to multiple potential species.

After integrating the synthetic CRISPR-Cas system into a strain of *P. aeruginosa* PA14 that had its native CRISPR-Cas deleted, and then confirming the synthetic system was fully functional against plasmids and the DMS3vir phage (electronic supplementary material, figure S1), we then created strains with two spacers each that both targeted one of 13 different phages. The panel of phages represented phylogenetically diverse origins, had a range of traits (being lytic and lysogenic), targeted different receptors, and displayed a range of values of infection dynamics (burst size, adsorption, eclipse period) and virulence (figure 2). This mirrors previous studies that have tested *P. aeruginosa* strains on a range of phages to investigate the evolution of spontaneous resistance mutations [53,54]. However, as we engineered each CRISPR-Cas-containing *P. aeruginosa* strain to already possess spacers against the phage, we were able to study the efficacy of CRISPR-Cas interference in isolation, unbiased by the likelihood of prerequisite spacer acquisition.

For eight of the 13 phages tested, CRISPR-Cas interference was able to provide complete protection of growth in liquid culture and significant decreases in titre when plating phages on lawns of each strain (figure 3; electronic supplementary material, figures S5–S12). This demonstrates that the measured phage characteristics could not be used to predict whether a CRISPR-Cas system could provide robust phage interference and protection to the host. For the lysogenic LBP1 (figure 3; electronic supplementary material, figure S14) and JBD63c (figure 3; electronic supplementary material, figure S13) phages, CRISPR-Cas interference provided intermediate protection in high-nutrient liquid growth conditions, but complete protection in low-nutrient conditions, thus showing that environmental parameters can affect the functioning of CRISPR-Cas interference.

Several previous studies have observed interactions between phage infectivity and nutrient conditions. For example, a study in *E. coli* investigating the maintenance of lytic lambda phage population in the presence of resistant cultures found that phages were only maintained in seven of 12 replicate populations in maltose-limited minimal medium but maintained in all replicates grown in high-nutrient both [55]. However, in a single-cell microfluidics experiment with *E. coli* and its phage T4, it was observed that increasing nutrients improved collective bacterial survival in the presence of phages [56].

Other work has suggested a link between growth rate and the evolution of CRISPR-Cas immunity via spacer acquisition. When populations of *P. aeruginosa* PA14 were cultured with DMS3vir in the presence of bacteriostatic antibiotics, spacer acquisition increased and evolution of CRISPR-Cas immunity was favoured over resistance via surface-modification [29]. This preference towards CRISPR-Cas immunity was also observed with growing the bacteria on carbon sources upon which growth was slowed [29]. This is in line with previous work showing that, when bacteria are grown in nutrient-limited media, evolution of phage resistance via CRISPR-Cas immunity is favoured over surface-modification-based resistance [26]. Slower growth through reduced temperature was shown to promote both CRISPR-Cas adaption and interference in *P. aeruginosa* PA14, including upregulation of the Cas complex [28]. These results seem to be general across bacteria; in *Pectobacterium atrosepticum*, *cas* gene expression was shown to increase upon deletion of the *galK* gene involved in galactose metabolism, suggesting a role for metabolic status in CRISPR-Cas regulation [57]. The metabolic status of the cell, as well as host density (which will be reduced in low-nutrient conditions) has also been shown to affect lysis–lysogenic choice in temperate phage [58], for example in poor media the lysogenic lifecycle dominates for lambda phage [59,60]. This could also interact with the efficacy of CRISPR-Cas interference.

Furthermore, phage dose and environmental conditions can impact the efficacy of *acr* genes possessed by a phage. Broadly the pattern of predicted Acr genes in the genomes of each phage does not correlate with the observed patterns of successful CRISPR-Cas interference (electronic supplementary material, figure S2), although these genes are predicted in both LBP1 and JBD63c. However, different *acr* only interfere with specific CRISPR-Cas system types. Previous studies have shown that phages in possession of *acr* genes are able to proliferate in CRISPR-Cas-carrying hosts at high phage titres but not at low phage titres owing to phage cooperation [61]. In addition to this, the addition of bacteriostatic antibiotics to the environment slows the translation of phage genes, enhancing the protection CRISPR-Cas systems are able to provide to the host against phages with Acrs [30].

Although both versions of the phage-targeting spacers against the JBD63c phage were functional when the protospacer was incorporated into a plasmid (electronic supplementary material, figure S13D), one version of the phage-targeting synthetic CRISPR-Cas strain in *P. aeruginosa* produced no protection from phage lysis, corroborating the importance of spacer choice [62]. Experiments in *E. coli* showed that when the CRISPR-Cas system was directed against T5 lytic phage, only spacers that targeted pre-early genes (which enter the cell first upon infection to shut down cell activity [18]) resulted in effective CRISPR-Cas interference, and spacers targeting other regions provided no protection despite being effective at plasmid targeting [19]. This previous study also saw that for two of the lytic phages used—T4 and R1-37—the CRISPR-Cas system in *E. coli* was unable to produce any protection against these phages [19]. This may have been due to DNA chemical modifications (which have been shown to inhibit CRISPR-Cas immunity [22]) or some other unknown mechanism. In the current study, two lysogenic phages, JBD18 (electronic supplementary material, figure S15) and JBD25 (electronic supplementary material, figure S16), were both unable to be targeted by CRISPR-Cas interference despite the spacers being functional against plasmids. This may have been caused the spacer choice (as seen for the phage JBD63c) or by another unknown mechanism. Given that JBD18 and JBD25 were described as ‘CRISPR sensitive’ in a previous study with the *P. aeruginosa* PA14 wild-type strain [20], the former spacer-based explanation is more likely. The synthetic CRISPR-Cas system also provided no protection against the jumbo phage phiKZ, which validates previous results that show PhiKZ forms a proteinaceous shell around its DNA in the host that can prevent the action of type I CRISPR-Cas systems [21,63].

The finding that CRISPR-Cas can only offer complete protection against lysis by some phages in low-nutrient conditions highlights the importance of considering the environment when testing phage infectivity and host range with defence systems. High-nutrient, high-growth environments are unlikely to reflect the ecological conditions of most bacteria–phage interactions. Other factors have also been shown to play a role in host infectivity; recently in *Acinetobacter baumannii* the host range of its phage Mystique was shown to vary between tests in liquid and solid media [64]. Similarly, in this study, we found that the protective effects of CRISPR-Cas immunity produced more pronounced phenotypes in liquid rather than solid media experiments. This may be due to the downregulation of CRISPR-Cas systems during surface-associated growth, which was described in a study on the regulation of CRISPR-Cas by alginate regulators in *P. aeruginosa* PA14 [65]. Understanding these layered and nuanced interactions between phages and defence systems will allow us to better understand how bacteria may be able to evade and overcome the use of phages in agricultural or therapeutic settings. Larger screens across more and diverse phages would facilitate searching for broad patterns and allow the identification of predictive characteristics in phages that correlate with CRISPR-Cas immunity.

## Data Availability

Sequences and experimental datasets available at Zenodo [66]. Supplementary material is available online [67].
